# Factor Structure of Catatonia in Catatonic Depression

**DOI:** 10.1192/j.eurpsy.2022.833

**Published:** 2022-09-01

**Authors:** A. Barkhatova, M. Bolgov

**Affiliations:** Mental Health Research Center, Department Of Endogenous Mental Disorders And Affective Conditions, Moscow, Russian Federation

## Abstract

**Introduction:**

Depression with catatonic features is a relatively common condition that can pose difficulties in nosological assessment and lead to life-threatening complications.

**Objectives:**

To determine the structure of catatonia associated with depression, and its subtypes.

**Methods:**

The exploratory factor analysis with maximum likelihood (MLE) data extraction and varimax rotation was used in a sample of 96 patients with depressive, bipolar or schizophrenia spectrum disorders, who were depressed and who met the criteria for catatonia according to the Bush-Francis Catatonia Screening Instrument (BFCSI).

**Results:**

The factor analysis revealed four factors of catatonia in depression, accounting for 57.3% of the variance. “Agitated” factor (eigenvalue 5.65, 18.2% of the variance) includes agitation, impulsivity, emotional lability, verbigeration, sudden muscular tone alterations, ambitendency, perseveration and stereotypy. “Hypokinetic” factor (eigenvalue 5.05, 16.3% of the variance) includes mutism, withdrawal, stupor, staring, negativism, rigidity, posturing and gegenhalten. “Proskinetic” factor (eigenvalue 3.65, 11.8% of variance) includes automatic obedience, mitgehen, echophenomena, catalepsy and waxy flexibility. “Parakinetic” factor (eigenvalue 3.41, 11.0% of variance) includes grimacing, flat affect, compulsive emotions, mannerisms and compulsive behavior. “Agitated catatonia” is a more specific subtype and is usually associated with bipolar disorder. “Hypokinetic catatonia” is the most common but less specific subtype. “Proskinetic catatonia” in depression does not occur apart from other subtypes of catatonia. “Parakinetic catatonia” is most commonly associated with schizophrenia spectrum disorders.

**Conclusions:**

Our study shows the heterogeneity of catatonic features in depression and facilitates the nosological diagnosis of catatonic depression.

**Disclosure:**

No significant relationships.

